# Impact of a Concurrent Respiratory Virus Infection on the Clinical Presentation and Response to Initial Treatment of Kawasaki Disease: A Single-Center Observational Study

**DOI:** 10.3390/jcm14030775

**Published:** 2025-01-24

**Authors:** Taichi Koyanagi, Ryuichi Nakagawa, Mari Okada, Haruna Yokoyama, Saori Amano, Teruyoshi Shimoyama, Tomohiro Udagawa, Natsuko Suzuki, Susumu Hosokawa, Masayuki Nagasawa

**Affiliations:** Department of Pediatrics, Musashino Red Cross Hospital, 1-26-1 Kyonan-cho, Musashino City 180-8610, Tokyo, Japan; baseball.taichi@gmail.com (T.K.); ryuichi_nakagawa_squall_raxhephon@msn.com (R.N.); okada-mr@musashino.jrc.or.jp (M.O.); haruna.y.mar@gmail.com (H.Y.); saori8187@yahoo.co.jp (S.A.); terustarrysky@gmail.com (T.S.); uda0112@yahoo.co.jp (T.U.); shosokawa.ped@tmd.ac.jp (S.H.)

**Keywords:** Kawasaki disease, multiplex polymerase chain reaction, respiratory syncytial virus, respiratory tract infections

## Abstract

**Background:** The impact of respiratory viral infections associated with Kawasaki Disease (KD) cases on KD’s clinical presentation and initial response to treatment has not been clearly determined. **Objective**: This study aimed to evaluate respiratory viral infections using FilmArray Respiratory Panel (FARP) testing and analyze the effect of the concurrent presence of pathogens on clinical presentations of KD. **Methods**: Between January 2021 and June 2023, we conducted a retrospective, single-center observational study of 105 Japanese children with KD. KD was diagnosed and treated according to RAISE study guidelines, and the cases’ clinical information was assessed. FARP testing was performed in 71 out of 105 KD cases with fever and/or respiratory symptoms. **Results**: In 38 (53.5%) out of 71 cases, at least one virus was detected. The FARP-positive cases tended to have a higher frequency of Kobayashi scores (K-scores) ≥ 5 than the negative cases (42.1% vs. 21.2%), and lower initial treatment failure (7.89% vs. 21.2%). The most common virus detected was rhino/enterovirus (RV/EV: 27 cases), followed by seven cases of respiratory syncytial virus (RSV). RV/EV-positive KD cases did not differ significantly in their clinical data or the frequency of K-scores ≥ 5, and RSV-positive cases showed significantly elevated liver enzyme (AST:59 U/L (43.5–150.5) vs. 35 U/L (27–41), ALT:40 U/L (28.5–244.5) vs. 18 U/L (14–27)) and CRP levels (12 mg/dL (7.3–14.2) vs. 6.5 mg/dL (4.1–8.5)), and an increased frequency of K-scores ≥ 5 (71.4% vs. 21.2%) compared to FARP-negative cases. KD cases that were also RSV-positive or RV/EV-positive showed favorable responses to initial treatments. **Conclusions:** Concurrent respiratory virus infection could affect the clinical manifestation and initial treatment response of KD.

## 1. Introduction

The Japanese physician Tomisaku Kawasaki initially documented the Kawasaki disease (KD) in 1967 [1]. An excessive immune response influenced by genetic factors, environmental elements, or infection triggers its onset; however, the exact pathophysiology remains elusive [2,3]. The incidence of KD exhibits patterns of outbreaks and seasonality, leading to speculation regarding infectious agents’ involvement, particularly respiratory infectious agents, such as adenovirus, streptococcus, and influenza viruses [4,5,6,7]. During the coronavirus disease 2019 (COVID-19) pandemic, reports from various countries, including Japan, have shown a decline in KD cases [8,9,10], which was attributed to the implementation of societal measures, such as lockdowns, universal masking, and thorough general infection prevention practices, resulting in reduced respiratory viral infections [11]. These epidemiological phenomena have prompted a reconsideration of the relationship between KD and respiratory viral infections.

Previous studies analyzing the association between KD and respiratory viral infections have suggested that the presence or absence of respiratory viral infections does not result in significant differences in the clinical features or complications of KD. However, reports analyzing the impact of concurrent “specific” respiratory viral infections on the clinical characteristics and responsiveness to treatment of KD are scarce. In this study, we utilized the FilmArray Respiratory Panel (FARP) test at the time of KD diagnosis to assess the status of respiratory viral infections and analyzed how the concurrent presence of pathogens affects the clinical manifestations of KD, focusing on their role beyond initial disease triggering. This study provides valuable insights into the complex interplay between KD and respiratory viral infections, shedding light on potential associations that warrant further exploration.

## 2. Methods

### 2.1. Patients

This study focused on a cohort of 105 Japanese children newly diagnosed with KD, following the Kawasaki Disease Diagnosis Guidelines, between January 2021 and June 2023 [12] at our institution, a tertiary emergency and core hospital in western Tokyo. All cases were admitted and received intravenous immunoglobulin (IVIG) therapy (2 g/kg) and oral aspirin (30 mg/kg/day) upon confirmation of the diagnosis [13,14,15]. Prednisolone (2 mg/kg/day) was administered alongside IVIG therapy in cases with a Kobayashi score (K-score) of 5 or higher, indicating potential resistance to initial IVIG treatment [16]. Cases who did not respond to the initial treatment received additional treatment according to the physician’s judgment. Resistance to initial treatment was defined as follows: no decline in fever (<37.5 °C) or re-elevated fever of 37.5 °C or more within 24–36 h after IVIG injection [16]. Coronary artery abnormalities (CAAs) were assessed using two-dimensional echocardiography without blinding and evaluated by z-score, where a z-score < 2.0 was considered normal, 2.5 > z-score ≧ 2.0 was considered dilatation, 5.0 > z-score ≧ 2.5 was considered a small aneurysm, 10 > z-score ≧ 5.0 was considered a medium aneurysm, and z-score ≧ 10.0 was considered a giant aneurysm [17]. In addition, we classified a CAA that normalized within 1 month as a transient dilation, and a CAA that remained after 1 month as an aneurysm [18]. Case evaluations included variables such as age at diagnosis, sex, date of diagnosis, blood test parameters, K-scores, responsiveness to initial treatment, and coronary artery abnormalities.

### 2.2. FARP Testing

Between January 2021 and June 2023, our institution conducted FARP testing at the physician’s discretion for newly admitted cases having a fever and/or respiratory infections as a part of COVID-19 infection control measures. Otherwise, the severe acute respiratory coronavirus 2 (SARS-CoV-2) quantitative antigen test was performed as an alternative. The FARP test (BioFire^®^ FilmArray Respiratory Panel 2.1, BioMerieux Japan, Tokyo, Japan) detects the nucleic acids of various pathogens in nasopharyngeal swab samples, identifying influenza viruses, coronaviruses, parainfluenza viruses, human metapneumovirus, adenoviruses, respiratory syncytial virus (RSV), human rhinovirus/enterovirus (RV/EV), *Mycoplasma pneumoniae*, *Chlamydia pneumoniae*, *Bordetella pertussis*, *Bordetella parapertussis*, and severe acute respiratory syndrome coronavirus 2 (SARS-CoV-2). Physicians used FARP as a diagnostic tool to swiftly identify respiratory pathogens in cases with fever and/or respiratory symptoms, aiding in timely disease management.

### 2.3. Statistical Analysis

The Mann–Whitney U test was used to compare non-normally distributed data between groups, while the chi-squared test or Fisher’s exact test was used to analyze categorical variables. Statistical significance was set at *p* < 0.05. All analyses used EZR software (ver 1.65), which is suitable for non-normally distributed and categorical data, ensuring robust and transparent results at a significance level of 0.05.

## 3. Results

**Figure 1:**
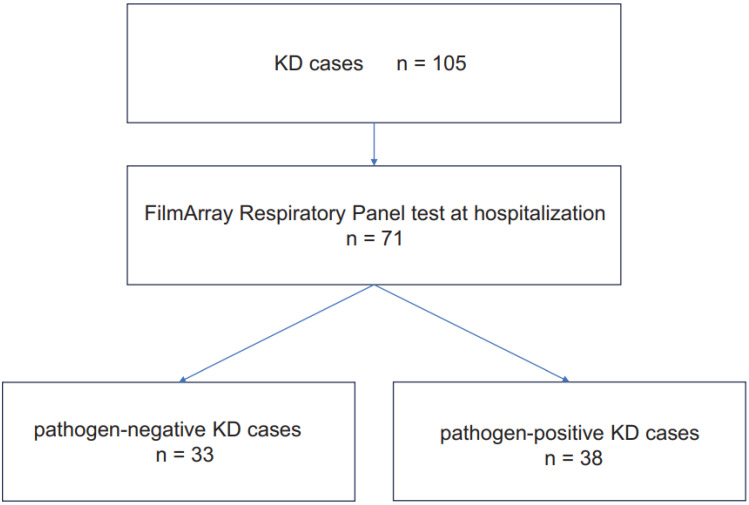
Flowchart of the stratification of cases with KD. We conducted FARP tests on 71 hospitalized cases with KD, classifying them into negative and positive groups. No cases were excluded during this process.

The FARP testing was conducted upon admission on 71 out of 105 cases hospitalized for KD. Among these, 38 cases tested positive for one or more viruses, whereas 33 tested negative for all viruses (Figure 1). The FARP test results are listed in Table 1. Two and three viruses were positive in six and two cases, respectively. Moreover, two cases were identified as co-positive for RV/EV and RSV, and singular cases of RV/EV were detected alongside parainfluenza virus type 4, parainfluenza virus type 3, SARS-CoV-2, and coronavirus NL63. Furthermore, two distinct combinations revealed triple viral positivity: one set with RV/EV, RSV, and SARS-CoV-2 and another featuring RV/EV, adenovirus, and parainfluenza virus type 4. The most frequently detected virus was RV/EV (27 cases, 38.0%). RV/EV was the sole positive virus in 19 cases, while the remaining eight cases were detected concurrently with other viruses. RSV was the next most detected virus, with seven cases (9.9%), of which three cases also tested positive for RV/EV. The frequency of virus detection in cases with KD (one or more positive cases: 53.5%; 38/71) did not significantly differ from the virus detection frequency in non-KD cases (one or more positive cases: 66.5%; 677/1018, *p* = 0.293) aged <5 years with acute respiratory infections tested using FARP during the same period at our institution (Table 1).

Next, we focused on KD cases who tested positive for RV/EV or RSV, as these were the most frequently detected viruses in our cohort. Table 2 compares the clinical characteristics between the two groups from the four cohorts: 38 KD cases with any virus detected (FARP-positive) vs. KD cases with no FARP detected (FARP-negative), 27 KD cases with RV/EV positivity (RV/EV-positive; including cases with co-positivity for additional viruses) vs. KD cases with FARP-negative, and 7 with RSV positivity (RSV-positive; of which three cases were co-positive for RV/EV) vs. KD cases with FARP-negative. When comparing the FARP-negative group with the FARP-positive group, aspartate aminotransferase (AST) levels were significantly elevated (*p* = 0.0454). No significant differences were observed in any clinical parameters between the RV/EV-positive group and the FARP-negative group. However, RSV-positive cases demonstrated significantly higher proportions of K-scores ≥ 5 (*p* = 0.008) and increased levels of AST (*p* = 0.0039), alanine aminotransferase (ALT) (*p* = 0.02), and C-reactive protein (CRP) (*p* = 0.03) compared to those in the FARP-negative group. The initial treatment failure rate was lower in the FARP-positive, RV/EV-positive, and RSV-positive groups than in the FARP-negative group, although the difference was not statistically significant. Cases with KD who were in the FARP-positive, RV/EV-positive, or RSV-positive groups did not exhibit any coronary artery abnormalities (CAAs). In our cohort, a CAA was observed in only one case (1.7%: 1/71), which was lower but not significantly different from our previous report [19] (2.7%: 6/225) in which prednisolone was not used for high-risk KD patients as an initial treatment.

When we focused on RV/EV-single-positive and RSV-single-positive cases with KD, the same tendency was observed (Table 3). No significant differences were observed between RV/EV-single-positive cases and FARP-negative cases. RSV-single-positive cases with KD (n = 4), despite the small sample size, showed higher levels of liver enzymes, elevated CRP, increased K-scores, and better responsiveness to initial treatment compared to FARP-negative KD cases.

In seven FARP-negative KD patients with K-scores ≥ 5, three were unresponsive to initial treatment, while all five RSV-positive KD patients with K-scores ≥ 5 were responsive to initial treatment.

## 4. Discussion

The exact cause of KD is not yet fully understood. However, it is thought to develop on the basis of an abnormal immune system response that results in the inflammation of blood vessels throughout the body, and a combination of environmental, infectious, and genetic factors are thought to be involved [20].

The human immune system is formed by a complex network. Its basic components are the innate immune system and acquired immunity; the latter matures after birth in the process of exposure to external stimuli, including pathogenic microorganisms such as respiratory viruses. The acquired immune system also forms a complex network, which can be simplified into a balance of T helper type 1 (Th1) cells for antiviral and antitumor immunity, Th2 cells for parasite immunity, Th17 cells that control neutrophil immunity to bacterial and fungal infections, and Treg cells that regulates those T helper cells [21,22]. Thus, infants with immature acquired immunity are subject to frequent respiratory viral infections.

In children hospitalized with respiratory viral infections who are under 5 years of age, the preferred age of KD, FARP testing detects one or more viruses in 70–80% of patients. Of these, multiple viruses are detected in approximately 30% of cases [23,24]. This indicates that children under 5 years of age are frequently infected by respiratory viral infections, and that children with KD are very likely to be affected by respiratory viral infections. A recent report from Japan showed that 65 out of 148 KD patients (43.9%) had positive FARP tests, and 204 out of 730 non-KD (28.0%) patients without respiratory symptoms had positive FARP tests during the period from April 2020 to September 2021 [25].

In this study, we focused on cases with KD who tested positive for RV/EV or RSV, and analyzed the impact of the concurrent presence of these viruses on the clinical characteristics of KD. In summary, our study demonstrated that cases with RSV-positive KD showed (1) a significant tendency towards liver dysfunction and elevated CRP levels, (2) an observed trend of higher K-scores, and (3) a lower rate in initial treatment failure compared with FARP-negative KD cases. On the other hand, when comparing RV/EV-positive KD cases with FARP-negative cases, we observed: (1) no significant differences in clinical laboratory data and K-scores, and (2) a lower rate of initial treatment failure in RV/EV-positive KD cases, although this difference did not reach statistical significance.

Although liver dysfunction is commonly observed in viral infections, the increase in CRP levels typically remains mild following RSV infection alone. Simultaneously, cases with RSV-positive cases in our cohort did not show any remarkable complications, such as wheezing/oxygen demand, or any other characteristic respiratory symptoms usually observed in RSV infection. The reason for a tendency towards elevated CRP levels and a lack of typical respiratory symptoms in cases with RSV-positive KD in this study is beyond the scope of the analysis and remains challenging to explain. A previous study indicated that KD cases with influenza infections had a tendency towards elevated CRP levels and that influenza, assumedly, plays a role in the inflammatory response [4]. Similar to influenza virus, RSV infection is considered to lead to inflammatory responses through the production of interferons and tissue necrosis factor [26].

In our study, the initial treatment failure rate was lower in the FARP-positive, RV/EV-positive, and RSV-positive groups than in the FARP-negative group, though the difference was not significant. Even in KD patients with K-scores ≥ 5, the initial treatment failure rate was lower in RSV-positive groups than in the FARP-negative group. The reason for this is not known, but there are two possibilities speculated. One is that there is some immunological difference between KD complicated by a respiratory virus infection and uncomplicated KD, and that this is modified by a concurrent respiratory virus infection. It has long been known that viral infection affects the immune status of the host. It is well known that in various immune diseases, viral infections are associated with a relapse, recurrence, or worsening of the underlying immunological diseases. On the other hand, improvement and remission of the immune disease state after measles or influenza B infection has rarely been reported [27,28,29].

In RV infections, nasal and bronchial epithelial cells are the first targets of RVs and are responsible for initiating anti-viral responses [30]. Viral recognition occurs via various pattern recognition receptors (PRRs), including retinoic acid-inducible gene-I (RIG-I)-like receptors (RLRs) and Toll-like receptors (TLRs), which induce the production of inflammatory mediators and interferons (IFNs). This inflammatory “cascade” induces the recruitment and activation of innate cells that can affect the phenotype of adaptive responses. RVs enhance interleukin-25 (IL-25), IL-33, and thymic stromal lymphopoietin (TSLP), which drive type 2 immune responses [31,32,33]. In healthy individuals, the primary immune responses to RV infection are Th1 and are characterized by a release of IFN-γ [34]. Th2 immune responses are characterized by the increased production of IL-4, IL-5, and IL-13, and have been associated with RV infection in asthma [30,32]. Another report presented that RV infection enhanced the Th2 environment by modulating programmed cell death ligand 1 (PD-L1) and PD-L2 expression levels in allergic mucosa and by increasing the IL-4/IFN-γ ratio in non-allergic mucosa [35]. Treg cell function might be altered or impaired during RV infections, which might play an important role in the association between RV and the development of asthma and asthma exacerbations [36].

In the context of our current study, the reported differences in initial treatment response rates based on K-score stratification [19,37,38] may be partially attributed to variations in respiratory viral infection complication rates among study cohorts.

Another is that intensified initial treatment is applied to KD complicated by respiratory virus infection due to higher risk scores. In our cohort, RSV-positive cases with KD showed a tendency for higher K-scores, leading to more frequent use of combination therapy with steroids. This might have potentially reduced the occurrence of treatment-resistant cases. It is considered that neutrophilic inflammation can be part of a harmful response, whereas CD8+ T cells and IFN-γ have protective roles, with a Th2-biased response being deleterious in the immune response to RSV infection [39]. It is interesting to note that the different types of viruses transmitted concurrently can have different effects on the responsiveness to treatment or clinical manifestations of KD. It is anticipated that the accumulation of such research findings will lead to the development of risk prediction scores for treatment responsiveness that take into account the coexistence of specific pathogens in KD in the future.

In our comparative analysis of cases with KD, no significant clinical differences were identified between FARP-negative and RV/EV-positive cases. A previous study also found no significant differences in clinical characteristics between RV/EV-positive and RV/EV-negative KD cases, suggesting that RV/EV does not exert a specific influence on the clinical presentation of KD [40]. In contrast, a detailed examination of the RSV-positive KD group clarified some of the previously discussed characteristics. Caution is necessary when interpreting these findings because of RV/EV co-positivity in some RSV-positive cases in our cohort. Nonetheless, the clinical characteristics of cases with RSV-positive KD predominantly reflect the impact of pure RSV infection rather than concurrent RV/EV infection because it is hypothesized that, as mentioned above, RV/EV does not yield any specific consequences on the clinical characteristics of KD. Furthermore, although the sample size was small, analysis of cases with RSV-single-positive KD consistently revealed significant clinical features, such as liver dysfunction, elevated CRP, and higher K-scores, supporting the hypothesis that RSV infection may play a unique role in the clinical presentation of KD.

Previous reports have conducted analyses on respiratory virus infections collectively in cases with KD and have suggested insignificant differences in the clinical features of cases with KD based on the presence or absence of respiratory viral infections [41,42], except for the slight difference in responsiveness to initial treatment between pathogen-negative and pathogen-positive cases with KD in one study [43]. This could be attributed to the aggregation of various viruses for analysis, blurring the individual characteristics of each virus and resulting in the absence of clinical significance. Conversely, our study is the first to specifically analyze the association between RSV infections and the clinical features of KD. The widespread adoption of the FARP test during the COVID-19 pandemic, enabling a detailed examination of respiratory viral infections, significantly influenced the results of this study. The findings of this study suggest that the FARP test contributes markedly to our understanding of the relationship between specific respiratory viruses and KD.

One limitation of this study is that viral RNA or DNA detection in respiratory samples may not necessarily reflect the current infection. Particularly in RV/EV, RNA can persist in nasal discharge for an extended period, and the possibility that RV/EV detected by FARP may have lower activity than RSV should be considered [44]. Confirming the current infection using antigen testing, in addition to FARP screening for positive cases, may enhance data accuracy. Another limitation is that this was a retrospective study conducted at a single institution with limited cases. Larger prospective studies are needed to reach conclusions, and further research is required.

## 5. Conclusions

In this study, RV/EV and RSV were identified as the most prevalent respiratory viruses among cases with KD. Especially, cases with KD who tested positive for RSV exhibited a tendency towards elevated liver enzyme and CRP levels, as well as increased K-scores. KD cases infected with RV/EV and/or RSV presented relatively favorable initial treatment responses compared to FARP-negative KD cases.

## 6. Key Points

By incorporating FARP testing, this study thoroughly analyzed the clinical presentation of KD in the concurrent presence of specific viral infections. Accumulating and analyzing cases for each virus individually may reveal more about the characteristics of KD in the presence of specific viral infections.

## Figures and Tables

**Table 1:** Viruses detected in KD and non-KD cases.

Pathogen	Number of Cases in KD (%)	Number of Cases in Non-KD (%)
RV/EV	27 (38.0)	398 (39.1)
RSV	7 (9.9)	188 (18.5)
PIV3	1 (1.4)	101 (9.9)
PIV4	1 (1.4)	6 (0.6)
AdV	2 (2.8)	82 (8.1)
HKU1	1 (1.4)	4 (0.4)
OC43	2 (2.8)	12 (1.2)
NL63	2 (2.8)	7 (0.7)
hMPV	1 (1.4)	57 (5.6)
PIV1	0	16 (1.6)
FluAH3	0	10 (1.0)
FluH1N1pdm	0	1 (0.1)
SARS-CoV-2	3 (4.2)	57 (5.6)
At least one or more positive	38 (53.5)	677 (66.5)
Double positive	6 (8.5)	165 (16.2)
Triple positive	2 (2.8)	34 (3.3)
Quadruple positive	0	7 (0.7)
Quintuple positive	0	2 (0.2)
Multiple positive	8 (11.3)	208 (20.4)

Data are presented as n (%). PIV3, parainfluenza virus type 3; PIV4, parainfluenza virus type 4; Adv, adenovirus; HKU1, human coronavirus; OC43, human coronavirus OC43; NL63, human coronavirus NL63; hMPV, human metapneumovirus; PIV1, parainfluenza virus type 1; FluAH3, influenza A virus subtype H3; FluH1N1pdm, influenza A(H1N1).

**Table 2:** Comparison of KD cases: FARP-negative vs. FARP-positive, RV/EV-positive, or RSV-positive.

FARP	Negative n = 33	Positive n = 38	*p*-Value	RV/EV (+) n = 27	*p*-Value	RSV (+) n = 7	*p*-Value
Sex (male/female)	20/13	27/11	0.45	20/7	0.41	5/2	0.63
Age (month, IQR)	33 (10–47)	26.5 (19.5–43.3)	0.96	28 (22–43.5)	0.95	15 (9–20.5)	0.29
Days since illness onset (day, IQR)	4 (3–5)	5 (4–6.8)	0.16	5 (4–6)	0.33	4 (3–6.5)	0.96
K-score ≥ 5	7 (21.2%)	16 (42.1%)	0.23	9 (33.3%)	0.26	5 (71.4%)	0.008 ^a^
K-score (IQR)	3 (2–4)	3 (1–5)	0.90	3 (1–5)	0.49	6 (4–6.5)	0.07
Resistance to initial treatment	7 (21.2%)	3 (7.9%)	0.17	2 (7.4%)	0.17	0 (0%)	0.18
WBC (/μL, IQR)	14,600 (11,500–15,500)	13,150 (11,550–15,885)	0.51	12,900 (11,000–15,150)	0.28	13,200 (1650–15,950)	0.75
Platelet (×10^4^/μL, IQR)	35.4 (26.9–42.5)	33.5 (27.8–42.1)	0.77	33.9 (27.5–42.1)	0.85	29.3 (25.9–35.5)	0.38
Na (mEq/L, IQR)	135 (133–136)	135.5 (133.3–136.8)	0.25	136 (135–136)	0.07	133 (131–135)	0.14
AST (IU/L, IQR)	35 (27–41)	41.5 (31.5–73.3)	0.04 ^b^	40 (33.5–72.5)	0.09	59 (43.5–150.5)	0.004 ^b^
ALT (IU/L, IQR)	18 (14–27)	24.5 (15–105.5)	0.12	22 (16–89.5)	0.15	40 (28.5–244.5)	0.02 ^b^
CRP (mg/dL, IQR)	6.5 (4.1–8.5)	6.6 (4.2–10.3)	0.55	5.0 (4.0–8.3)	0.66	12.0 (7.3–14.2)	0.03 ^b^
CAA	1 (3.0%)	0	0.28	0	0.36	0	0.64

Data are presented as n (%) unless otherwise indicated. WBC, white blood cell; Na, serum sodium. ^a^ *p* < 0.05 as determined by Fisher’s exact test when compared to FARP-negative cases. ^b^ *p* < 0.05 as determined by the Mann–Whitney U test when compared to FARP-negative cases.

**Table 3:** Comparison of KD Cases: FARP-negative vs. RV/EV-single-positive and FARP-negative vs. RSV-single-positive.

FARP	Negative n = 33	RV/EV (+) n = 19	*p*-Value	RSV (+) n = 4	*p*-Value
Sex (male/female)	20/13	5/14	0.01 ^a^	3/1	0.58
Age (month)	33 (10–47)	32 (23.5–47.5)	0.58	20.5(16.3–27.5)	0.70
Days since illness onset (day)	4 (3–5)	5 (4–6)	0.3	5 (3.8–6.3)	0.63
K-score ≥ 5	7 (21.2%)	6 (31.5%)	0.45	4 (100%)	0.001 ^a^
K-score	3 (2–4)	3 (1–5)	0.3	6 (5.8–6.8)	0.02 ^b^
Resistance to initial treatment	7 (21.2%)	1 (5.2%)	0.09	0 (0%)	0.57
WBC (/μL)	14,600 (11,500–15,500)	13,100 (11,950–14,700)	0.4	14,250 (12,725–16,100)	0.85
Platelet (×10^4^)	35.4 (26.9–42.5)	37 (28.0–39.7)	1.0	29.2 (26–33.8)	0.261
Na (mEq/L)	135 (133.0–136.0)	136 (134.5–136)	0.07	131 (129.5–132.3)	0.02 ^b^
AST (IU/L)	35 (27.0–41.0)	37 (31–64)	0.31	51.5 (43.5–101.8)	0.003 ^b^
ALT (IU/L)	18 (14–27)	21 (14.5–89.5)	0.45	118.5 (22.5–228.3)	0.17
CRP (mg/dL)	6.5 (4.1–8.5)	4.96 (3.7–7.1)	0.26	13 (11.0–14.1)	0.01 ^b^
CAA	1 (3.0%)	0	0.45	0	0.72

Data are presented as n (%) unless otherwise indicated. WBC, white blood cell; Na, serum sodium. ^a^ *p* < 0.05 as determined by Fisher’s exact test when compared to FARP-negative cases. ^b^ *p* < 0.05 as determined by the Mann–Whitney U test when compared to FARP-negative cases.

## Data Availability

Data are available upon request from the authors.
